# The prediction of the risks of spontaneous combustion in underground coal mines using a fault tree analysis method

**DOI:** 10.1016/j.mex.2024.102835

**Published:** 2024-07-03

**Authors:** Mustafa Emre Yetkin, Muharrem Kemal Özfırat, Mete Kun, Cagatay Pamukcu

**Affiliations:** Mining Engineering Department, Dokuz Eylul University, Izmir, Buca 35160, Turkey

**Keywords:** Spontaneous combustion, Coal mining, Risk analysis, Fault Tree Analysis (FTA), Method of prevention of spontaneous combustion

## Abstract

Mining is one of the most risky and dangerous sectors. It is impossible to ignore the losses of life and material experienced by occupational accidents, which take place in the field of mining. Risk analysis begins with a risk assessment to identify the probability and severity of workplace hazards. Hazards must be controlled by precautions according to the risk score levels.

In this study, a fault tree analysis method was conducted to analyze spontaneous combustion hazards and to predict future risks in underground coal mines. Three main causes of the top event were defined and for each of these causes, risk scores were computed using a fault tree analysis. Finally, the causes of spontaneous combustion, which is an event that is frequently encountered in coal mines, were discussed, and the spontaneous combustion risk probability was calculated as 0.3012 in cases of air entry into the gob and failure to prevent coal-air contact in development drifts. As a result of the study, the fundamental causes of spontaneous combustion, the greatest hazard in underground coal mining worldwide, have been examined in detail. The innovative approach introduced by the study aims to increase the awareness and recognition of conditions that lead to spontaneous combustion among industry workers and engineers through detailed evaluation. By doing so, it seeks to minimize the occurrence of spontaneous combustion incidents.

• This paper introduces a main flowchart and countermeasure algorithm to prevent spontaneous combustion.
• This paper also analyzes events which trigger spontaneous combustion and mentioned preventive measures for this events.

## Graphical abstract

**Figure:**
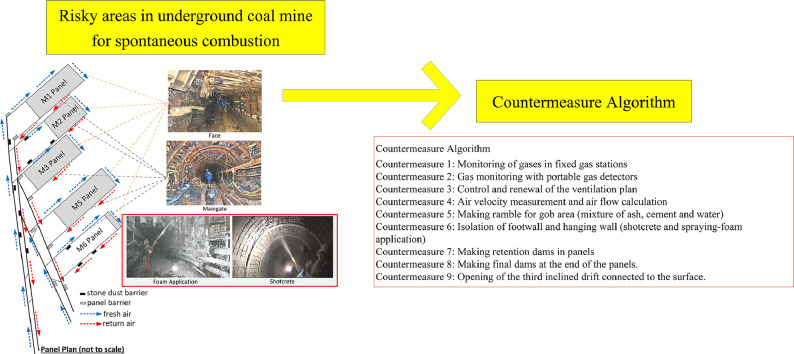

Specifications table

**Table:** 

Subject area:	Engineering
More specific subject area:	*Mining Engineering*
Name of your method:	*Method of prevention of spontaneous combustion*
Name and reference of original method:	*N/A*
Resource availability:	*N/A*

## Background

Endogenous mine fires are those that occur by internal heating. Formation of such fires does not involve introduction of heat from outside but the main cause is the heat stemming from oxidation. The accepted theory about spontaneous combustion of coal is based on absorption of oxygen (oxidation) by coal under suitable atmospheric conditions. There is an exogenous reaction between the coal and the oxygen [1,2].

As soon as surfaces of coal contact air, oxidation starts. In a mine under normal conditions, the exhausted heat is evacuated, and the oxidation process is maintained in a slow pace without the danger of any heating. However, in some cases, the exhausted heat cannot leave its environment, and the temperature in this environment constantly increases. If there is enough oxygen in the environment, oxidation speed increases at increased temperatures, and the temperature of the coal also increases in turn. When the ignition temperature (critical temperature) of coal is reached, combustion occurs [3].

**Fig. 1:**
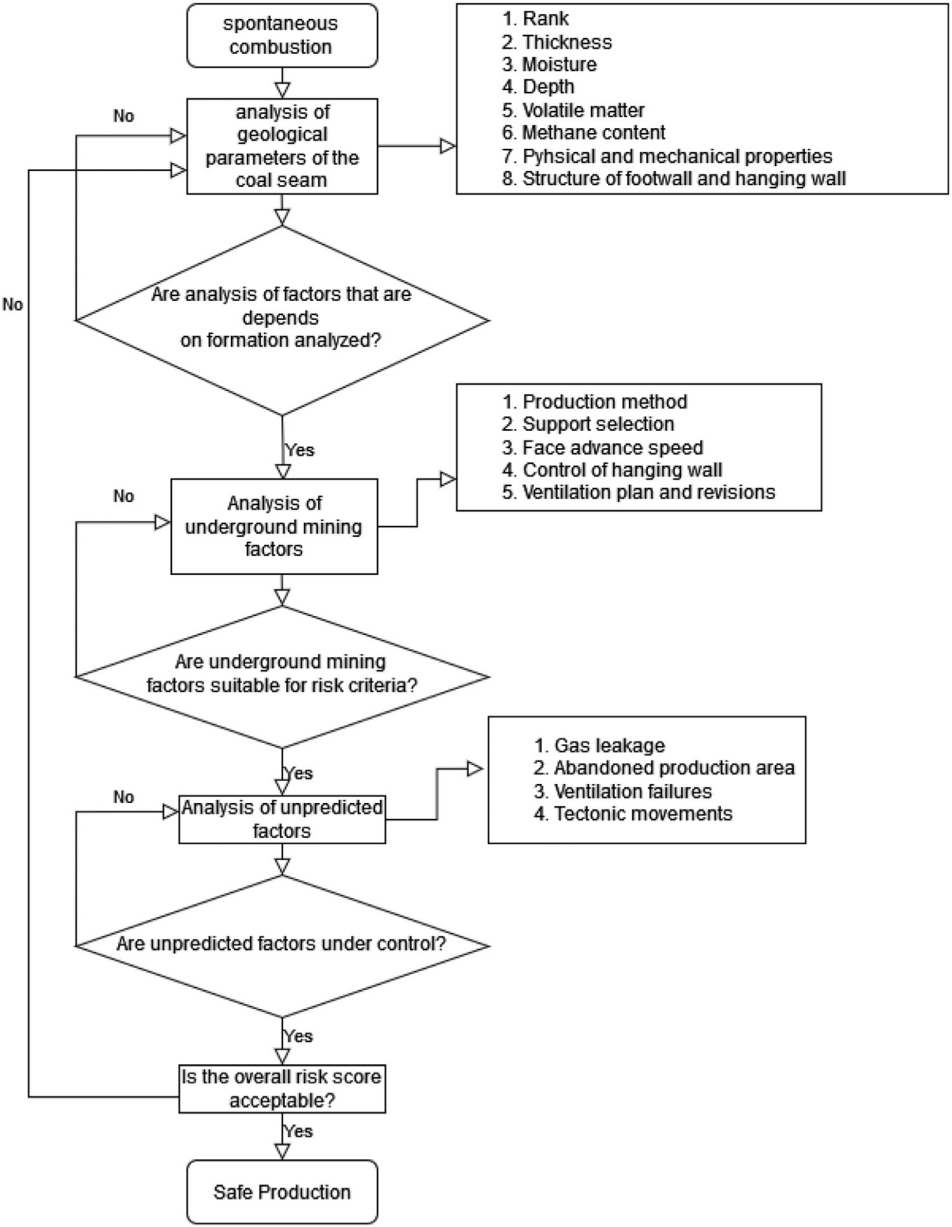
Main flowchart in spontaneous combustion.

In this study, the probability values of hazards calculated by methods of fault tree analysis which could lead to fires, provided a main flowchart consisting of all parameters towards preventing spontaneous combustions. This way, the main parameters that could lead to coal fires were reduced to the lowest level of risk by usage and control of the suggested main flowchart (Fig. 1).

In recent years, deaths occurring as a result of underground coal mining have increased, due to serious accidents in different places of the world. These accidents are mostly based on firedamp explosions and spontaneous combustion of the coal. Toxic gasses are formed as a result of spontaneous combustion and many fatal accidents may happen due to exposure of employees to these gasses. The spontaneous combustion of coal is a serious event that must be taken into consideration since it prevents production and adversely affect occupational health results in underground mines [[4], [5], [6], [7], [8], [9], [10], [11], [12]]. There are many test methods that have been used to probe spontaneous combustion in coal, but these methods need a long time in order to determine spontaneous combustion before it occurs. There is creates a need for a rapid and reliable method to test coal undergoing self-heat in the coal industry. Some new methods are being developed by scientists to predict spontaneous combustion areas using numerical analysis, temperature and gas monitoring in longwall working areas [5,10,[13], [14], [15], [16], [17], [18], [19], [20], [21]].

In this study, hazards that may lead to spontaneous combustion in underground coal mines were identified and classified using a fault tree analysis method. According to classified hazards, counter measures in order to prevent spontaneous combustion were determined. In addition, this study aims to fill the gap in literature and field studies by integrating fault tree analysis into spontaneous combustion problem. Fault tree analysis and endogenous fire is an important motivation of the study. The study comprehensively addresses one of the most hazardous situations in coal mining: spontaneous combustion, along with all potential root causes. The goal is to increase the awareness of the parameters that lead to such incidents. By using the risk analysis method, these root parameters are thoroughly evaluated, and the aim is to explain to all engineers and sector employees how these risks can be minimized. The study seeks to fill the gap in the sector's perspective on highly dangerous incidents and to prevent potential hazardous situations through increased awareness.

## Method details

### Fault Tree Analysis (FTA) methodology

FTA is a logical structured process which can help determine potential causes of system failure before the failures occur [22]. In an effort to carry out FTA, firstly, undesired events and a top event are needed to be examined. In the determination of the top event, the causes are determined classifying faults and sub-faults. After the build-up of fault tree, all branches linked to and/or gates and minimal cut set are found and evaluated for the top event.

In recent years, many researchers studied on FTA and its application to different areas. FTA was used to examine roof collapse, gas outburst event and spontaneous combustion in coal mines [[23], [24], [25], [26]], investigated the reasons of roof fall accidents in underground coal mines using FTA method with fuzzy logic. FTA was also used to analyze fatal accidents in open pit mining transportation systems and risks in underground tunneling applications in several studies [[27], [28], [29]], provides a general overview of fault tree analysis and examines its applications in model-based reliability analysis. Additionally, it discusses the advantages and limitations of fault tree analysis method and explores how it can be used in different scenarios. [21] assesses the factors contributing to spontaneous combustion in coal mines across South Africa. It examines geological conditions, mining practices, and mitigation strategies to manage and reduce the risk of spontaneous combustion incidents.

### Case study: Soma/eynez coal field-Turkey

**Fig. 2:**
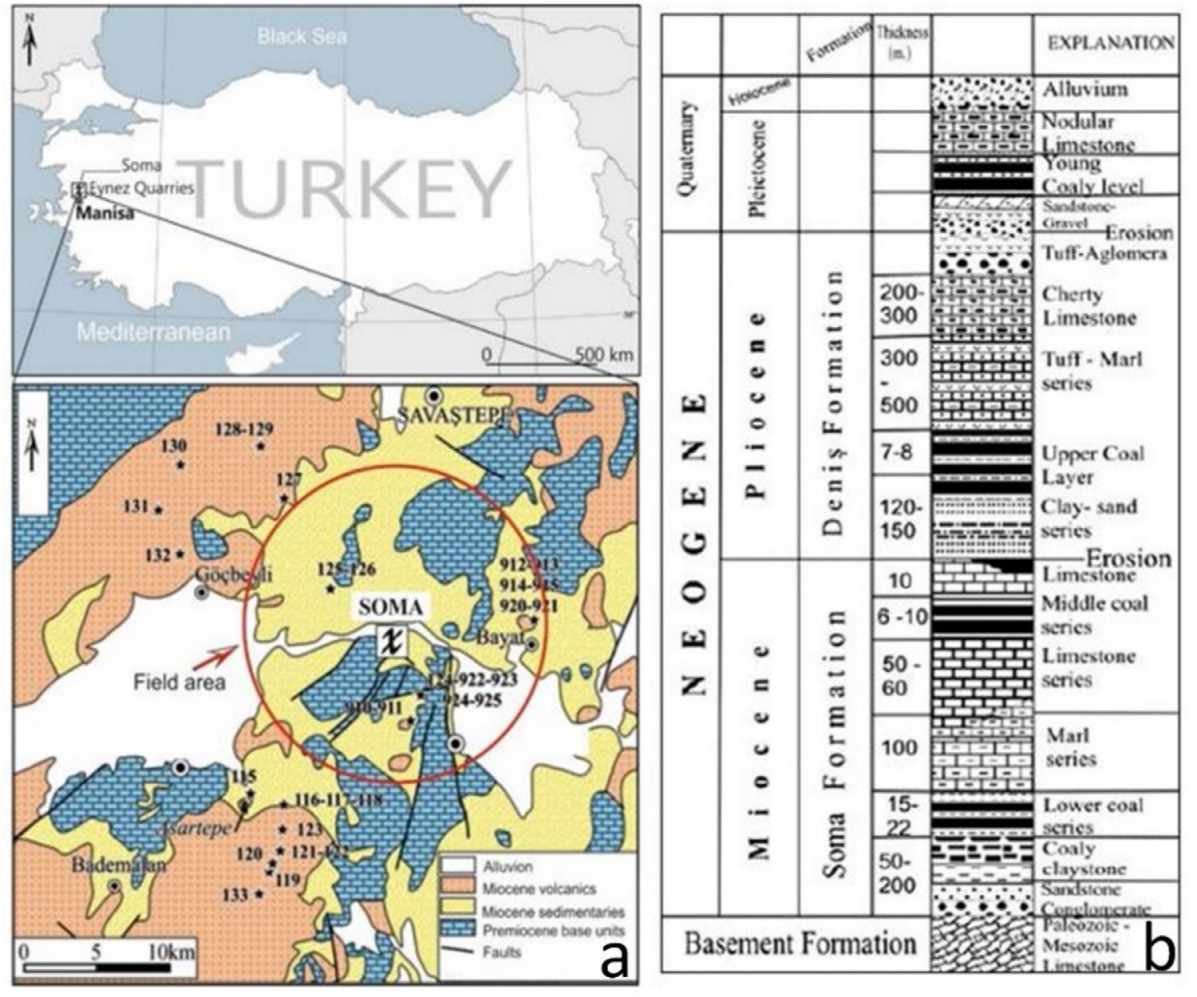
(a) Location and geological map of Soma and surroundings, (b) general stratigraphic columnar section of the study field [30].

In terms of the spontaneous combustion property of coal and the requirements of this combustion process, it is known that the main reason for the process of spontaneous combustion is the contact between coal and air. In regards to the determination of the points where coal contacts air, these points were marked as problematic, and a fault tree was started. When spontaneous combustion is examined, the gob behind a longwall, development drifts that opened the coal seam and abandoned mining spaces were determined as the areas where spontaneous combustions occurred. The location and geological map of the Soma and surroundings are given in Fig. 2.

The second stage involved determination of the oxygen sources that are required for fire to occur in these areas. In previous studies, experiences and past accidents that were reviewed, it was determined that several reasons such as air entry into the gob during ventilation, surface subsidences, faults and joints, contact between the surface and air during ventilation in development drifts and selecting blow-type ventilation systems directly or indirectly cause spontaneous combustion.

### Application of the study

Development of a fault tree usually involves six main steps. These are selecting a top-level event, combining known causes, forming the fault tree, evaluation, assessment of additional tests and results and reaching conclusions. In the fault tree analysis, the top-level event was taken as spontaneous combustion, and the sub-events which started it, were defined as air leak into the gob, air leak in development drifts and surface settlements or faults. According to SGK [31] data, 11 fatal accidents occurred in the mining sector with a total production volume of 640 million tons in 1100 mining enterprises with 53,000 employees. In the same year, 90 million tons of production volume was realized in 443 mining enterprises with 36,000 employees and 13 fatal accidents occurred. Between 2010 and 2020, 17 serious mining accidents occurred in Turkey [32].

**Fig. 3:**
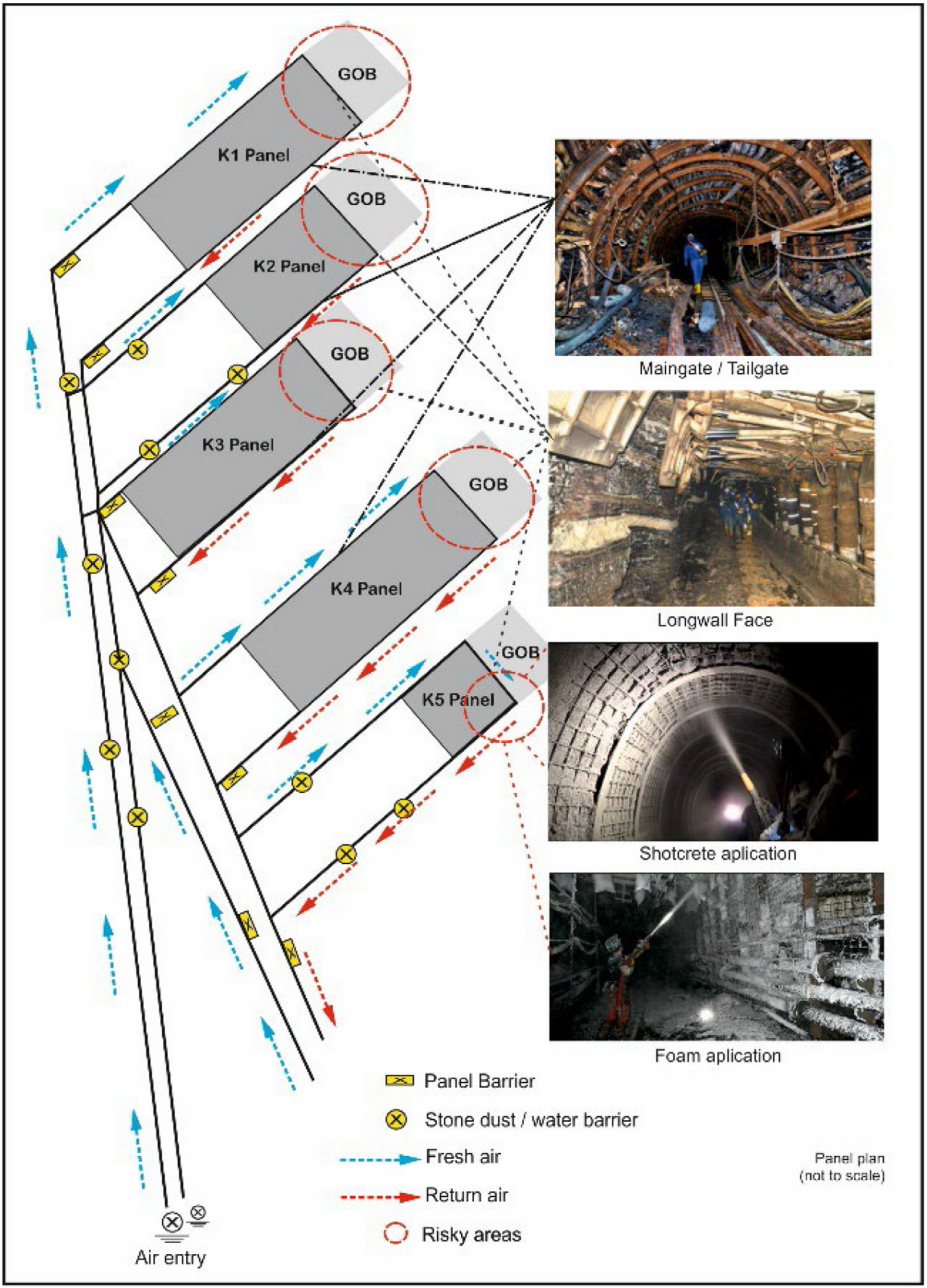
Risky areas in underground coal mine for spontaneous combustion.

**Fig. 4:**
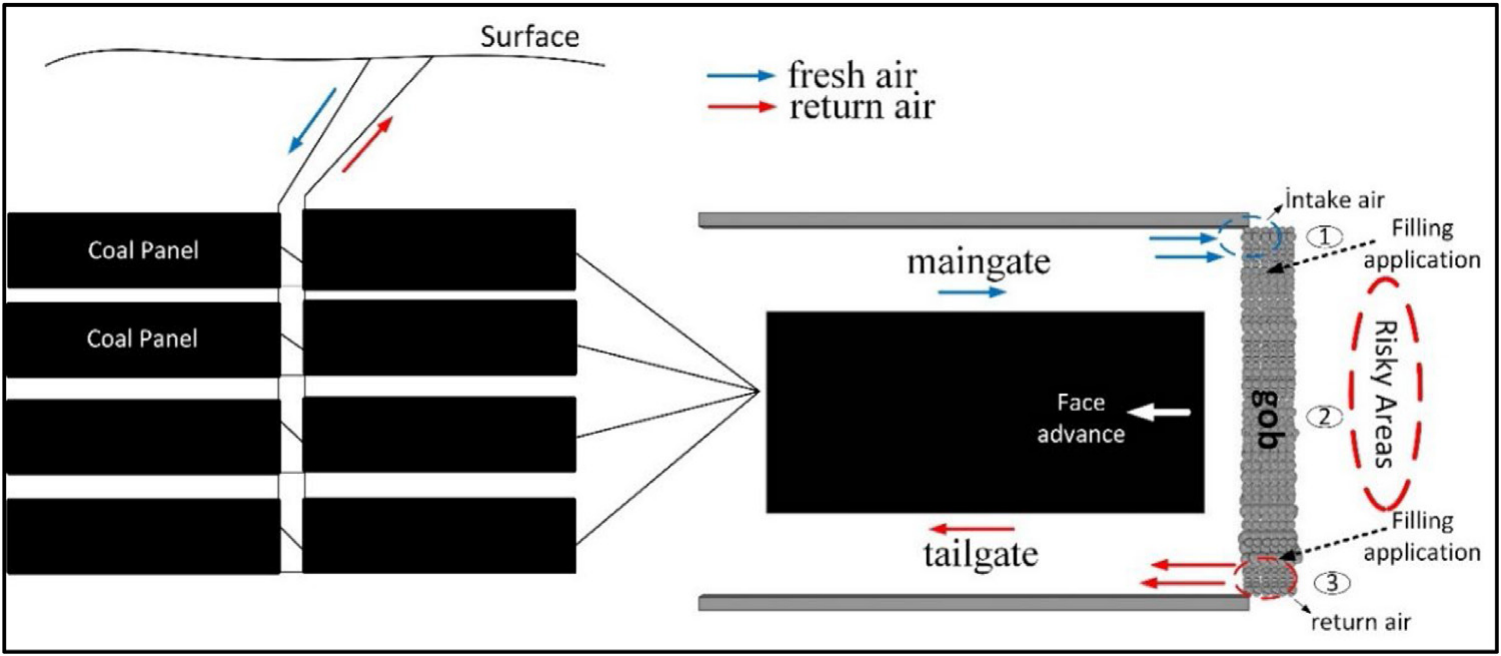
High risk areas under study in a longwall panel.

The reasons for these definitions were collected under three main categories namely developing drifts into the coal seam, formation of the gob behind a longwall and abandoned mining spaces. These events were accepted as the main branches of the fault tree, and the reasons for occurrence of events were defined under sub-branches. Fig. 3 shows the risky areas as an example on a mine plan in the Soma/Eynez region in Turkey. The areas, which have spontaneous combustion risks, are shown in Fig. 4.

The primary reason that may lead to fires during the development of drifts in the coal seam is non-isolated or improperly isolated drifts. The second main reason is leakage of air into gobs. These leakages are induced by stowed or non-stowed operations. The third main reason for fires is occurrence of combustion in abandoned places. These fires are directly related to the presence of fire dams. Normally, it is preferred that all preparation galleries are driven on the footwall in coal mines in terms of minimizing the surface deformation effect. Footwall in Eynez area is very soft claystone. While the thickness of clay (footwall coal) is low in the eastern parts of the field, it is quite high in the western part. Eynez and Ömerler coalfields are influenced by immense tectonic activities leading to the formation of adverse geological conditions for underground production [33]. The important point here is to reduce the contact of the air passing through the preparation galleries with the coal as much as possible. For this reason, the coal surface should be covered with non-flammable chemical foams. Foam, a gas-liquid dispersed system, holds significant importance and is typically produced using a foaming device through physical foaming. This innovative approach combines the fire protection benefits of yellow mud grouting and nitrogen injection technology effectively. It can also serve as a carrier to transport solid-phase materials and water to the coal seam simultaneously, enhancing its resistance effect [[34], [35], [36]]. Many endogenous fires were encountered during the production works in Eynez field. From this, it can easily be concluded that the risk of spontaneous combustion of coal is high. Another issue is that the coal of the Soma field is thick coal seam. One of the most critical issues in the production of thick coal seam is the fire problem, since the production area cannot be left immediately and the lower slices must be produced later [37,38]. In addition, in other similar coal seam samples in the world, researchers have studied the risk of spontaneous combustion of coal due to coal material structure, coal seam properties and geologic factors [[39], [40], [41], [42], [43], [44]].

[43] studied the endogenous fire properties of Soma coals in 2020. Calculations were made using the following equations in the measurements using the crossing point temperature method. Spontaneous combustion tendencies of coals were determined by using the data obtained during the experiment. Empirical equation used by [45] was used when making susceptibility classification (Eqs. (1) and (2)). The formulation of the mentioned index and the explanations of the variables used in the formulation are given below.

(1) $$I_{FCC}=AverageHeatingRate\left(AHR\right)/CrossingPointTemperature\left(CPT\right)\times 1000$$

Where;

I_FCC_: Feng, Chakravarty, Cochrane index, min^-1^ .

AHR: Average Heating Rate between 110 and 220 °C, °C min^-1^.

Average heating rate (AHR) is calculated by the formula given below;

(2) $$AHR=110/\left(t_{2}-t_{1}\right)$$

Where; t_2_: time when the coal sample reaches a temperature of 220 °C, min t_1_: time when the coal sample reaches a temperature of 110 °C, min

By using the index (I_FCC_), the spontaneous combustion tendencies of coals are classified according to I_FCC_ (I_FCC_: 0–5 low risk, I_FCC_: 5–10 medium risk, I_FCC_ >10 high risk). I_FCC_ values of Soma region mines ranged from 10.87 - 26.28 min^-1^, 14.6 - 28.78 min^-1^, 14.27 - 30.3 min^-1^ accordingly, Ilica et al., 2020 found Soma Basin lignite coals “high risk” in terms of spontaneous combustion.

**Fig. 5:**
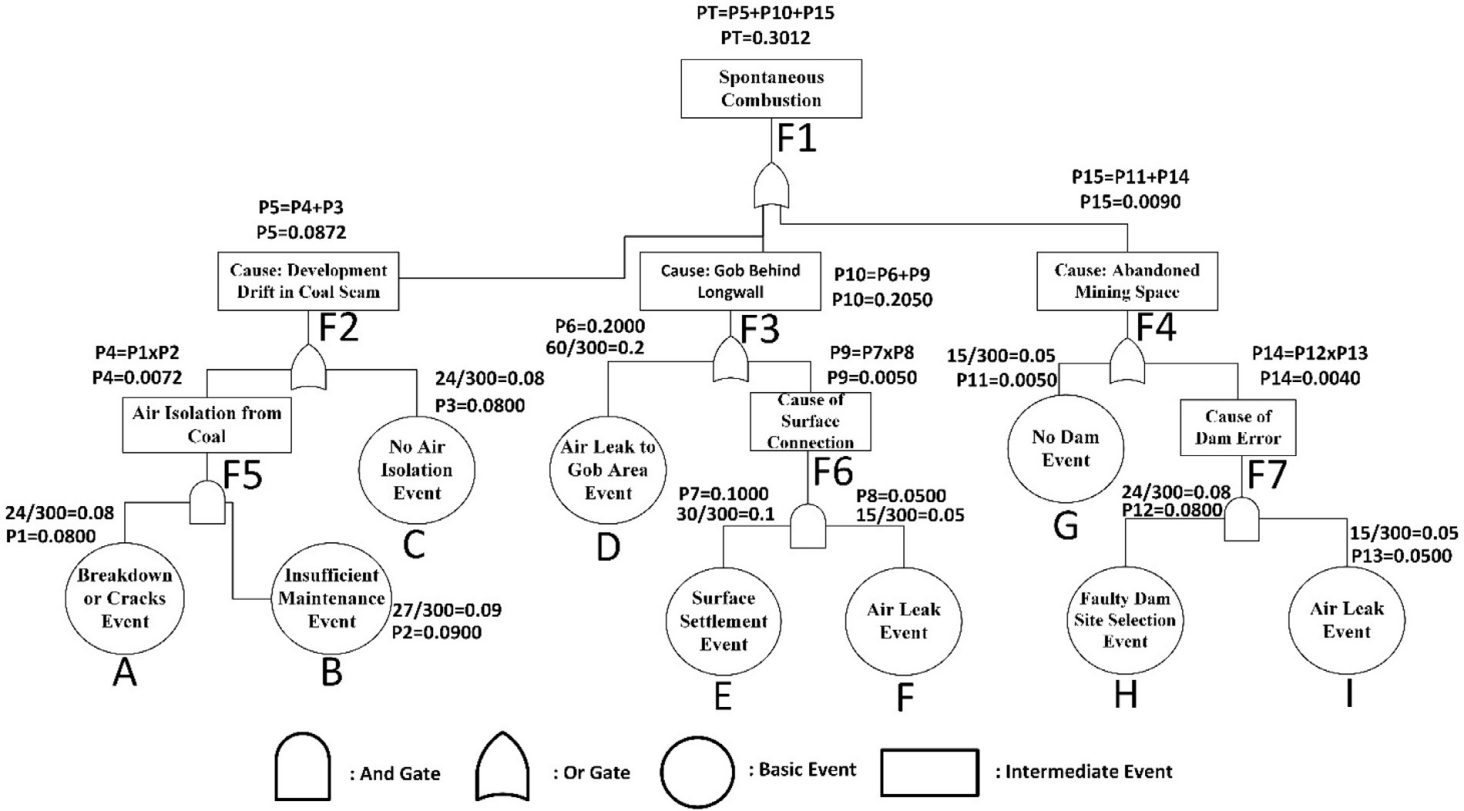
Fault tree model for spontaneous combustion in underground coal mines.

In order to quantify the events that may lead to a fire, codes were assigned to these as: spontaneous combustion (F1), opening development of drifts inside the coal (F2), the gob behind longwall (F3), abandoned mining spaces (F4), isolation errors (F5), surface connection (F6), dam errors (F7), breakdown or cracks (A), lack of machine maintenance (B), non-isolated drifts (C), air leakage into the gob (D), connection between surface settlement or a fault line and the gob (E), air leak (F), no fire dams (G), failure to eliminate contact with air (I) and incorrect selection of dam site (H). This way, it was aimed to make the structure created within the fault tree more comprehensible and to conduct the minimum cut set calculations faster. The spontaneous combustion fault tree composed for this study is illustrated in Fig. 5.

### Cut set research with equation reduction method

The purpose of cut set research in fault tree analyses is to define the system, determine the weaknesses of the system and make the system successful. In this analysis method, the top-level event is coded as “F1”, the simpler events connected to the top-level event are coded as “F2-F7”, the causing events are coded as “A-I” and probability is coded as “P”. The fault tree that is formed as a result of this coding process is shown in Fig. 5.

The calculations for the fault tree are given in the following equations.

(3) F_1_ = F_2_ + F_3_ + F_4_

(4) F_2_ = C+F_5_

(5) F_3_ = D+F_6_

(6) F_4_ = *G*+F_7_

(7) F_5_ = *A* x B

(8) F_6_ = *E* x F

(9) F_7_ = *H* x I

(10) F_T_ = C + (*A*×*B*) + D + (*E*×*F*) + *G* + (*H*×*I*)

(11) *P_j_* = NP_j_ / WD

Where;

P_j_: Probability of problem j

NP_j_: Number of days lost due to problem j (*j* = *A*, B, C, D, E, F, G, H, I)

WD: Number of working days in one year (assumed to be 300 days)

**Fig. 6:**
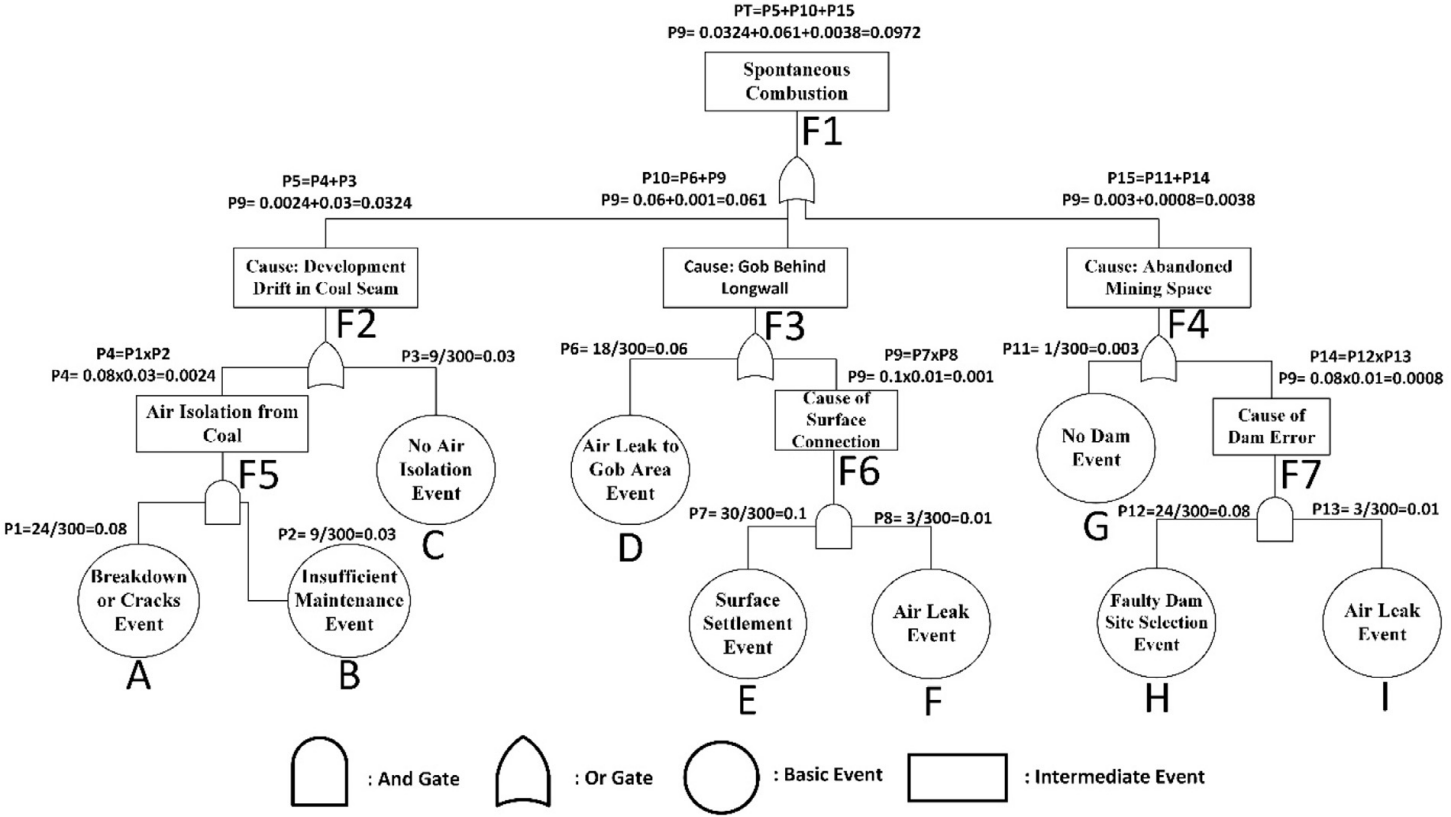
Probability calculation of spontaneous combustion.

For point A: PA = 24/300 = 0.08 (refer to Fig. 6)

For point B:PB = 27/300 = 0.09 (refer to Fig. 6)

PC, PD, PE, PF, PG, pH, PI values are computed similarly.

**Fig. 7:**
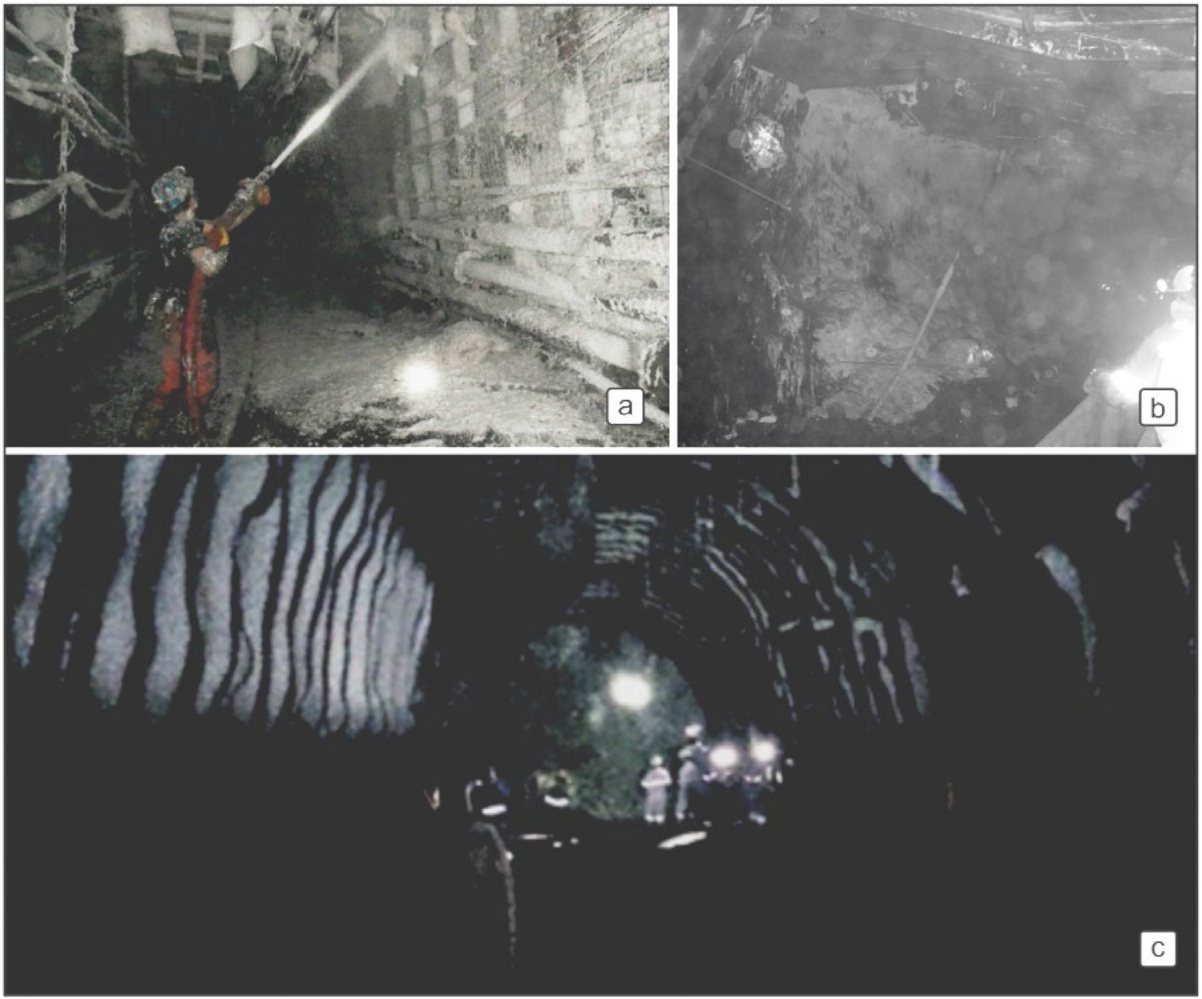
Spraying-foam (a-b) and shotcrete applications in the study area (c).

After precautions are taken;

Equal probability for point A: PA = 24/300 = 0.08 (refer to Fig. 7)

Decreasing probability for point B: PB = 9/300 = 0.03 (refer to Fig. 7)

The occurrence probabilities of the events that are selected were determined by utilizing the views of expert engineers working at mining companies, the experiences that are obtained from the literature and relevant project studies, production and ventilation reports of the mines, the fire events which occurred in the sector and their outcomes. The probabilities of the aforementioned events were statistically analyzed. According to official data, 13,852 deaths occurred due to work accidents in Turkey from 2010 to 2020. It is stated that 1042 of these deaths occurred in the mining sector. 304 of the fatal accidents are caused by underground fire [32]. Spontaneous combustion continues to be a hazard for U.S. underground coal mines, particularly in western U.S. where the coal is generally of lower rank. For the period 1990–2006, a total of 25 reported fires for underground coal mines in the U.S. were caused by spontaneous combustion [6]. In probability scores calculation, Eq. (11) was used. Here, NP_j_ is number of days lost due to problem and WD is number of working days per year. The fault tree was transformed into the form of Eq. (10) by calculations based on Boolean mathematics, and the final probability scores were calculated.

The three main causes that may trigger the event of spontaneous combustion are the development of drifts in the coal seam, the gob region behind longwall and failure to take precautions for abandoned mining spaces in Fig. 5. These three faults were divided into sub-branches within themselves. The probability score of the fault of opening development drifts in the coal (P5) was calculated as 0.0872. In this branch of the fault tree, the causes of risks that may be related to opening development drifts in the coal were examined. As such causes, lack of isolation in the development drifts (P3), collapses and shear-related cracks in the isolation (P1), damages due to different causes (P4) and failure to repair damages (P2) were examined. The probability scores of these risks were calculated as P1 = 0.08, P2 = 0.09, P3 = 0.08 and P4 = 0.0072. The probability score of the fire risks caused by the gob region behind the longwall (P10) was calculated as 0.205. In this branch of the fault tree, the problems encountered in practice are studied in places where the production method is longwall mining.

The greatest problems which are observed in non-stowed mining operations (P6) are leakages into the gob from the air that is transferred to the face for ventilation purposes. These leakages lead to fires by contacting coal in the gob. The other problems are subsidences that reach the surface (P9), non-thick collapses in seams appearing after production (P7) and contact of the gob with air (P8) igniting mine fire. The probability scores of these risks were found as P6 = 0.2, P7 = 0.1, P8 = 0.05 and P9 = 0.005, respectively. The main difference that separates these events from others is that detection, measurement and intervention in the events that take place in the gob take a long time, and they are high-cost operations. The probability of a fire risk caused by abandoned mining spaces (P15) was calculated as 0.009. In this branch of the fault tree, the problems that could be caused by these issues were examined. Those problems might be counted as the failure to construct the dam to shut down the parts where production is completed (P11) or failure to construct it properly (P14). These risks are faulty dam site selection (P12) and air leakage (P13) risks. The probability scores of these risks were calculated as P11 = 0.005, P12 = 0.08, P13 = 0.05 and P14 = 0.004. Fig. 5 shows these scores on the composed fault tree. This way, it becomes easier to see the connections of faults between each other.

Probabilities are re-calculated as in Fig. 6 after improvement measures. As a result of the improvement studies, it has been observed that the possibilities have decreased significantly. These improvements can be summarized as follows. In order to prevent the contact of the coal behind the longwall with the air, filling process just after production, increasing the production speed as much as possible, making gas measurements regularly, regular monitoring of the temperature in the mine and regular maintenance of the mine gas sensors, the characteristics of the coal and detailed study of the methane monitoring and drainage. In addition, search and modeling the geology of the mine, making continuous control in the risky regions in order to prevent air leakages, making the location selection of the dams by taking the comments and opinions of project and field engineers, reducing the surface settlements to minimum levels and preventing air coal and gob zones are other important measures. In risky areas to prevent coal and air contact, providing coal-air insulation with non-combustible chemical foam and continuous monitoring and control of abandoned mine sites will be possible to prevent from spontaneous combustion in mine.

## Method validation

In previous studies, various researchers reported on causes of coal fires in longwall mining. The main causes reported in such studies included air leakage, oxygen transport, heat transfer and exothermic reaction [7,8,10,11,16]. In this study, these causes were examined in a fault tree analysis, and the probability rates of the main sources of faults in coal fires were calculated. As a result of these calculations and the main causes, this study comprised similar results to those in other studies [4,5,9,12,[46], [47], [48], [49], [50]]. Additionally, studies by [5,10,[14], [15], [16], [17]] were utilized, and in eligibility for the fault tree model, the places where spontaneous combustions would occur and causes of occurrence were determined. Considering that the annual production of coal in Turkey is between 65 and 70 million tons according to projections, the annual coal production in coal fields, for example, in the Soma field, has reached 15 million tons. 10 million tons of this production is obtained from underground resources. In large amounts of production, the coal seams with thicknesses ranging from 15 to 35 m trigger fire events during production. Therefore, the factors that affect spontaneous combustion of coal include formation of coal, its chemical characteristics, employed production methods, ventilation systems and practices, rank of the coal, humidity and volatile matter content.

Due to the thickness of coal seams, longwall production processes with slicing is more difficult than in thin seams. In production processes, during the recovery of thick seams by collapsing method, the coals remaining behind the face may lead to fires. After reviewing previous studies [38,51] by fault tree analysis, the main parameters that need to be considered for preventing fires in mines were examined, and the 9 precautions were determined. These are monitoring of gasses in fixed gas stations, gas monitoring with portable gas detectors, control and renewal of the ventilation plan, air velocity measurement and air flow calculation, making ramble for gob area (mixture of ash, cement and water), isolation of footwall and hanging wall (shotcrete and spraying-foam application), making retention dams in panels, making final dams at the end of the panels, opening of the third inclined drift connected to the surface. In the stage of applying pre-determined precautions, Fig. 7 shows applications of spraying-foam and shotcrete which are employed the in study area to prevent spontaneous combustion risk.

As a result of taken measures and corrective / preventive applications as given in Fig. 7, the probabilities of the identified faults were recalculated using Eq. (11) and evaluated using Eq. (10). Accordingly, the newly calculated probability values are given in Table 1.

**Table 1:** Probability scores before and after measures.

Event code	Probability before measures	Probability after measures
PT	0.3012	0.0972
P1	0.0800	0.0800
P2	0.0900	0.0300
P3	0.0800	0.0300
P4	0.0072	0.0024
P5	0.0872	0.0324
P6	0.2000	0.0600
P7	0.1000	0.1000
P8	0.0500	0.0100
P9	0.0050	0.0010
P10	0.2050	0.0610
P11	0.0050	0.0030
P12	0.0800	0.0800
P13	0.0500	0.0100
P14	0.0040	0.0008
P15	0.0090	0.0038

## Conclusion and future research avenues

Spontaneous combustion is a significant risk for underground coal mines. This risk should be analyzed, followed, planned and managed in detail. The purpose of this study was to emphasize this risk and to give a detailed methodology for carrying out risk analysis. This may assist to prevent serious accidents in the mining industry. In this study, three main causes linked to twelve causes were examined for minimizing the risk of spontaneous combustion and to providing a safe working environment for Soma/Eynez underground coal mines. Each node is very important in the fault tree analysis of the endogenous fire problem. Errors to be made at these points will increase the probability of the top event occurring. In particular, the increase in the number of lost days with the problems and the intersection or merger of the errors will increase the possibility of endogenous fire. For this reason, reducing the number of lost days at each node with safety work together with the necessary precautions will reduce and eliminate the possibility of endogenous fire problem. Particularly, the coal of the region has high risk in terms of tendency to spontaneous combustion due to coal, geological properties, thick seam production. After the precautions in the fault tree, the number of lost days has been reduced and the probability of the endogenous fire problem has been reduced from 0.3012 to the level of 0.0972.

## Limitations

NA.

## Ethics statements

Not applicable.

## CRediT authorship contribution statement

**Mustafa Emre Yetkin:** Conceptualization, Methodology, Software, Validation, Data curation, Writing – original draft, Visualization, Investigation, Writing – review & editing. **Muharrem Kemal Özfırat:** Conceptualization, Methodology, Software, Validation, Data curation, Writing – original draft, Visualization, Investigation, Supervision. **Mete Kun:** Validation, Data curation, Writing – original draft, Visualization, Investigation, Supervision. **Cagatay Pamukcu:** Validation, Data curation, Writing – original draft, Visualization, Investigation, Supervision.

## Declaration of competing interest

The authors declare that they have no known competing financial interests or personal relationships that could have appeared to influence the work reported in this paper.

## Data Availability

Data will be made available on request.
